# Enhanced Photocatalytic Oxidative Coupling of Methane over Metal-Loaded TiO_2_ Nanowires

**DOI:** 10.3390/molecules30020206

**Published:** 2025-01-07

**Authors:** Shuang Song, Jiongcan Xiang, Hui Kang, Fengming Yang

**Affiliations:** 1 Institute of Fundamental and Frontier Sciences, University of Electronic Science and Technology of China, Chengdu 610054, China; ssabigale@gmail.com (S.S.); xjc952492@163.com (J.X.); 2 Institute of Advanced Study, Chengdu University, Chengdu 610106, China; 3 College of Computer Science and Cyber Security (Pilot Software College), Chengdu University of Technology, Chengdu 610059, China

**Keywords:** photocatalysis, oxidative coupling of methane, TiO_2_ nanowires, mild O_2_ adsorption and activation, active oxygen species

## Abstract

The photocatalytic oxidative coupling of methane (OCM) on metal-loaded one-dimensional TiO_2_ nanowires (TiO_2_ NWs) was performed. With metal loading, the electric and optical properties of TiO_2_ NWs were adjusted, contributing to the improvement of the activity and selectivity of the OCM reaction. In the photocatalytic OCM reaction, the 1.0 Au/TiO_2_ NW catalyst exhibits an outstanding C_2_H_6_ production rate (4901 μmol g^−1^ h^−1^) and selectivity (70%), alongside the minor production of C_3_H_8_ and C_2_H_4_, achieving a total C_2_–C_3_ hydrocarbon selectivity of 75%. In contrast, catalysts loaded with Ag, Pd, and Pt show significantly lower activity, with Pt/TiO_2_ NWs producing only CO_2_, indicating a propensity for the deep oxidation of methane. The O_2_-TPD analyses reveal that Au facilitates mild O_2_ adsorption and activation, whereas Pt triggers excessive oxidation. Spectroscopic and kinetic studies demonstrate that Au loading not only enhances the separation efficiency of photogenerated electron–hole pairs, but also promotes the generation of active oxygen species in moderate amounts, which facilitates the formation of methyl radicals and their coupling into C_2_H_6_ while suppressing over-oxidation to CO_2_. This work provides novel insights and design strategies for developing efficient photocatalysts.

## 1. Introduction

As worldwide demand for crude oil increases, methane, with high availability from natural gas, shale gas, biogas, and combustible ice, is regarded as an attractive alternative for fuel and a critical building block for producing chemical commodities [1,2,3]. However, the selective activation and conversion of methane remains a great challenge due to its inert nature, including high C-H bond dissociation energy (439 kJ mol^−1^), symmetrical geometry, and low polarizability, as well as high reactivity required for the desired products, which would require substantial energy input to overcome the thermodynamic barriers and increase the conversion [4,5]. Traditionally, the industrialized route for the commercial-scale transformation of methane has typically been achieved through indirect reforming methods like steam reforming (SRM) followed by syngas conversion, operating at high temperatures (700–1100 °C), and it is not only energy-intensive, but also accompanied by CO_2_ emissions [6]. The thermocatalytic oxidative coupling of methane (OCM) can realize the direct conversion of methane into C_2_ products with favorable selectivity at high temperatures, but this process is burdened by drawbacks comprising high investment in equipment, the deactivation of catalysts under harsh conditions, and the generation of undesired CO_x_, thus bringing about a barrier to commercialization [1,6,7,8,9]. Consequently, academia and industry have strong financial and environmental incentives to develop an efficient process that transforms methane into more valuable chemicals and fuels.

Photocatalysis, employing photons instead of heat, has emerged as a promising approach for methane conversion under mild conditions. Photons possess a special function to break the limitation of thermodynamic equilibrium, leading to a remarkable enhancement in yield [3,5,10,11,12]. C_2_H_6_, an important chemical raw material in modern industry, can provide a guarantee for the synthesis of succeeding compounds [13]. Recent advances have been made in both the photocatalytic oxidative and non-oxidative coupling of methane (OCM and NOCM) to ethane. In the non-oxidative system, Xiong et al. [14] fabricated a photocatalyst based on TiO_2_ by modifying the valence band with Pd-O_4_ units, which achieved a high C_2_H_6_ production rate of approximately 910 μmol g^−1^ h^−1^ after 3 h of light irradiation. Long et al. [15] achieved a high C_2_H_6_ production rate of approximately 11 μmol g^−1^ h^−1^ at optimized Au/ZnO. Although oxide-based photocatalysis provides opportunities for NOCM, it suffers from unsatisfying selectivity and the rapid decay of photoactivity, potentially stemming from lattice oxygen consumption or an excessive accumulation of holes within the semiconductors [14,16]. Alternatively, photocatalytic OCM, an appealing method, is not thermodynamically constrained and can achieve high C_2_H_6_ yield, selectivity, and stability. Recently, Tang et al. [17] upgraded photocatalytic OCM over a ternary Ag-AgBr/TiO_2_ catalyst in a pressurized flow reactor with an ethane yield of 35.4 μmol h^−1^, in which Ag nanoparticles functioned as an electron acceptor to improve charge separation, whereas holes could migrate from TiO_2_ to AgBr, preventing overoxidation. Yu et al. [18] demonstrate a photochemical looping concept for methane conversion to ethane at ambient temperatures under irradiation using Ag-HPW/TiO_2_, achieving a C_2_H_6_ yield of 20.7 μmol h^−1^ g^−1^ with an approximate selectivity of 80%.

Notably, the role of TiO_2_ in the aforementioned photocatalysis is extremely important. With stable physicochemical properties, a strong redox capability, earth abundance, low cost, and nontoxicity, TiO_2_ is widely studied and used in photocatalysis; however, the further application of TiO_2_ is hindered by the wide energy gap and remarkable recombination of charges [19]. Extensive research has been conducted to enhance the separation of photogenerated charge carriers via the design of nanostructures, such as one/two/three/zero-dimensional (1/2/3/0D) structures [20,21]. Among all of the TiO_2_ nanostructures, 1D morphology provides the semiconductor platform with a high surface area, increasing the amount of surface catalytic active sites, and, more importantly, 1D TiO_2_ nanowires (NWs) possess unique physicochemical properties and anisotropic structures that facilitate facile charge transport along the longitudinal dimension and accelerated separation of electron–hole pairs [22,23,24,25]. In addition, it has been demonstrated that the metal cocatalysts loaded on the surfaces of semiconductors can serve as electron traps to accept photogenerated electrons and can mostly tune the reaction pathway for the dominating generation of desired products [26,27,28]. Our recent work demonstrated that remarkable ethane production and selectivity could be realized in the photo-oxidation of CH_4_ with O_2_ over 1.0 Au-ZnO/TiO_2_ [29]. Inspired by these phenomena, exploring metal-modified TiO_2_ nanowires is a necessity.

Herein, we constructed an Au-modified anatase TiO_2_ nanowire photocatalyst (1.0 Au/TiO_2_ NWs) via a solvothermal synthesis, followed by subsequent calcination and a simple NaBH_4_ reduction method. The obtained 1.0 Au/TiO_2_ NWs photo-oxidized CH_4_ to C_2_H_6_ with O_2_ in a gas flow reactor effectively and selectively, giving rise to a high C_2_H_6_ production rate (4901 μmol g^−1^ h^−1^) with high selectivity (70%). TiO_2_ nanowires promote the separation of photoinduced charge carriers, while Au NPs facilitate the desorption of methyl (CH_3_) species to form •CH_3_ radicals in the gas phase, boosting the subsequent formation of C_2_H_6_.

## 2. Results and Discussion

**Figure 1:**
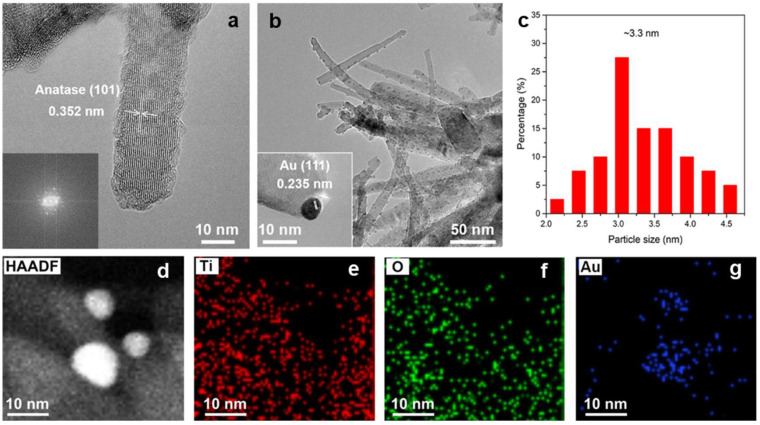
(**a**) HRTEM image of TiO_2_ NWs. (**b**) TEM image of 1.0 Au/TiO_2_ NWs. (**c**) The distribution of Au particle size on the surface of TiO_2_ NWs. (**d**) HADDF-STEM image of 1.0 Au/TiO_2_ NWs and the corresponding elemental maps for Ti, O, and Au (**e**–**g**) (scale bar: 10 nm). The inset of (**a**) is selected area electron diffraction (SAED). The inset of (**b**) is HRTEM of 1.0 Au/TiO_2_ NWs.

The SEM images (Figure S1) and STEM image (Figure S2) of TiO_2_ NWs show the morphology and the structure of one-dimensional anatase TiO_2_ nanowires with lengths of ca. 150 nm and diameters of ca. 10 nm. The HRTEM image of the related position is demonstrated in Figure 1a, indicating the high crystallization of TiO_2_ nanowires with the inter-plane spacing of 0.352 nm, which conforms to the (101) planes of anatase TiO_2_. From the low-resolution TEM image Figure 1b, the Au particles are uniformly dispersed on the surface of TiO_2_ NWs. Figure 1c shows that the mean size of Au nanoparticles dispersing on the surface of TiO_2_ NWs was less than 5 nm. The obvious crystallization lattice fringes with the inter-plane spacing of 0.235 nm was in accordance with the (111) planes of Au (inset in Figure 1b). The HADDF-STEM image (Figure 1d) and those elemental maps (Figure 1e–g) of Au/TiO_2_ NWs confirm its elementary ingredients of Ti, O, and Au, and reveal the further-dispersed situation of Au.

**Figure 2:**
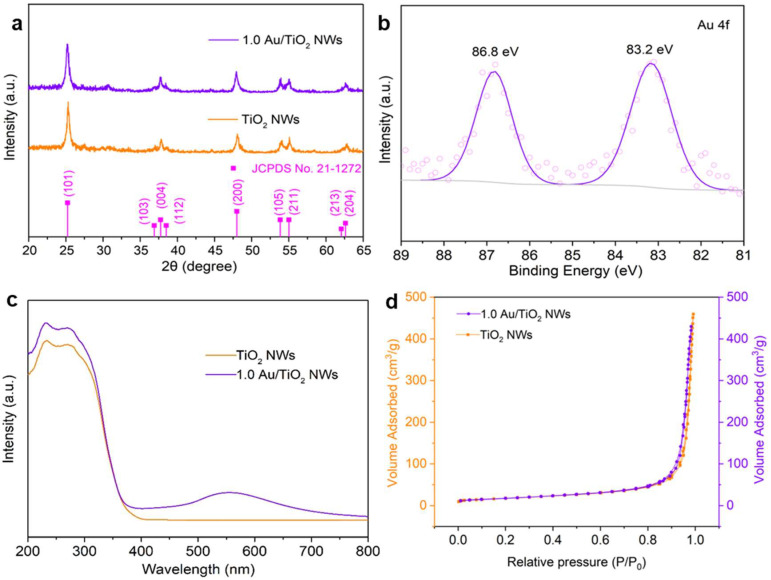
(**a**) XRD patterns of TiO_2_ NWs and 1.0 Au/TiO_2_ NWs. (**b**) XPS spectra of 1.0 Au/TiO_2_ NWs for the Au 4f spectrum. (**c**) UV-vis diffuse absorption spectrum of TiO_2_ NWs and 1.0 Au/TiO_2_ NWs. (**d**) N_2_ adsorption–desorption isotherms of TiO_2_ NWs and 1.0 Au/TiO_2_ NWs.

The crystal structures of TiO_2_ NWs and 1.0 Au/TiO_2_ NWs are substantiated by X-ray diffraction (XRD) analysis (Figure 2a). There are no other diffraction peaks except for the typical diffraction peaks of anatase TiO_2_ (JCPDS card no.21-1272). Additionally, compared with TiO_2_ NWs, the patterns for other samples lay out identical diffraction peaks without any diffraction peaks for Au, Ag, Pd, and Pt (Figure S3a). This is probably on account of their small size and highly dispersed situation on the surface of 1D TiO_2_ NWs. In order to analyze the surface atomic information of Au/ TiO_2_ NWs, XPS spectroscopy was used. The spectra of Au/TiO_2_ NWs were calibrated by fitting C 1s peak to 284.6 eV (Figure S4) in all situations. In refined Ti 2p XPS spectra of TiO_2_ NWs, bimodal peaks appear at 458.5 and 464.1 eV, which conforms to Ti 2p_1/2_ and Ti 2p_3/2_, respectively (Figure S5a). The Au 4f binding energy (83.2 eV) is in the region of Au^0^ (Figure 2b), indicating that Au particles deposited on anatase TiO_2_ NWs are metallic. According to UV-visible absorption spectra in Figure 2c, Au loading does not influence the bandgap and adsorption edge of anatase TiO_2_ nanowires. However, due to the plasmon resonance of Au particles, there is a significant increment of adsorption intensity for visible light at the range of the longer wavelength from 480 nm to 600 nm, especially within the scope of 530–540 nm. In Figure 2d, the specific surface areas of TiO_2_ NWs and 1.0 Au/TiO_2_ NWs are 61.5 m^2^ g^−1^ and 60.3 m^2^ g^−1^, respectively, which manifests that Au loading has no evident effect on specific surfaces (Figure S6).

**Figure 3:**
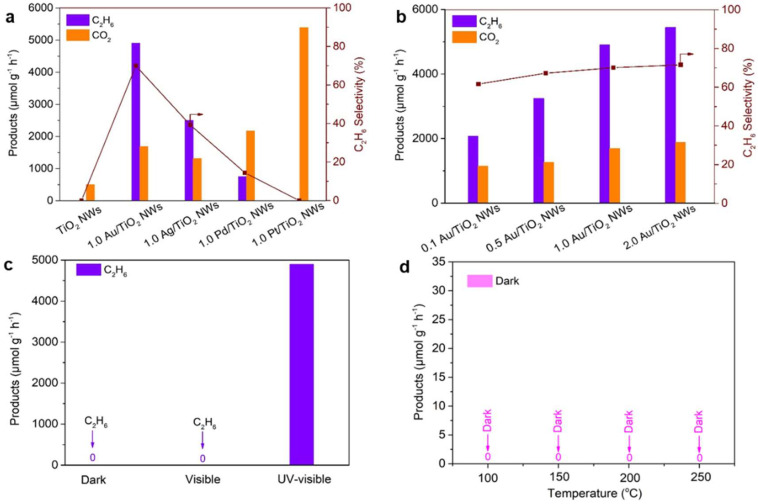
Photocatalytic OCM performance. (**a**) Photocatalytic activity of OCM over TiO_2_ NWs loaded with different cocatalysts. (**b**) Photocatalytic activity of OCM over TiO_2_ NWs loaded with different amounts of Au. (**c**) The contrast experiments performed under dark, visible and UV-visible conditions, respectively. (**d**) The contrast experiment performed under different temperature without light. Reaction conditions in (**a**–**d**): 20 mg photocatalyst, 69 mL min^−1^ of CH_4_ + 1 mL min^−1^ of air (20 vol.% O_2_/N_2_).

In total, 1.0 Au/P25, 1.0 Au/TiO_2_ (Rutile), and 1.0 Au/TiO_2_ (anatase, nanoparticle) were synthesized (Figure S7). Compared with 1.0 Au/P25 and 1.0 Au/TiO_2_ (Rutile), 1.0 Au/TiO_2_ NWs possessed superior performance in the OCM reaction (Figure S8). Therefore, the TiO_2_ NWs-based catalysts were much more suitable for the photocatalytic OCM reaction. Only CO_2_ was produced over the TiO_2_ NW catalyst, indicating the overoxidation of CH_4_. The metal catalysts have a crucial effect on yield and selectivity. The activity of 1.0 Au/TiO_2_ NWs displayed higher C_2_H_6_ production (4901 μmol g^−1^ h^−1^) and selectivity (70%). In addition, small amounts of C_3_H_8_ (351 μmol g^−1^ h^−1^) and C_2_H_4_ (15 μmol g^−1^ h^−1^) were also produced on 1.0 Au/TiO_2_ NWs, and the selectivity towards C_2_–C_3_ hydrocarbons (C_2_H_6_, C_2_H_4_, and C_3_H_8_) reached approximately 75%. Compared to the photocatalyst loaded with the Au cocatalyst, the C_2_H_6_ production rate of 1.0 Ag/TiO_2_ NWs decreased by about 49%, reaching only 2504 μmol g^−1^ h^−1^, with a C_2_H_6_ selectivity of 39%. The C_2_H_6_ production rate of 1.0 Pd/TiO_2_ NWs further dropped to 755 μmol g^−1^ h^−1^, with a selectivity of C_2_H_6_ only 14%. Surprisingly, in the photocatalytic OCM reaction involving 1.0 Pt/TiO_2_ NWs, only CO_2_ was detected as the product, with no other products observed. This indicates that the catalyst completely oxidized CH_4_ into CO_2_ (Figure 3a), while the conversion rate of 1.0 Au/TiO_2_ NWs is 0.45%, which is much closer to that of 1.0 Pt/TiO_2_ NWs (around 0.43%).

As the Au content increased from 0.1 wt.% to 1.0 wt.%, the C_2_H_6_ production rate and selectivity continuously improved due to the enhanced separation efficiency of photogenerated electrons and holes. However, when the loading was further increased to 2.0 wt.%, the activity only increased by 13%, while the selectivity remained essentially unchanged (Figure 3b and Table S1). Compared with 1.0 Au/TiO_2_ NWs, the high gold loading in 2.0 Au/TiO_2_ NWs might inhibit UV absorption, which likely explained the lack of significant improvement in its activity (Figure S9). To demonstrate that the photocatalytic OCM reaction was driven by light rather than the thermal effect induced by the surface plasmon resonance (SPR) of Au, a Y48 filter was employed to block ultraviolet (UV) light (Figure 3c). Under heating conditions without light, neither C_2_H_6_ nor CO_2_ was detected, further confirming that the OCM reaction in this study is driven by solar energy rather than thermal energy (Figure 3d).

**Figure 4:**
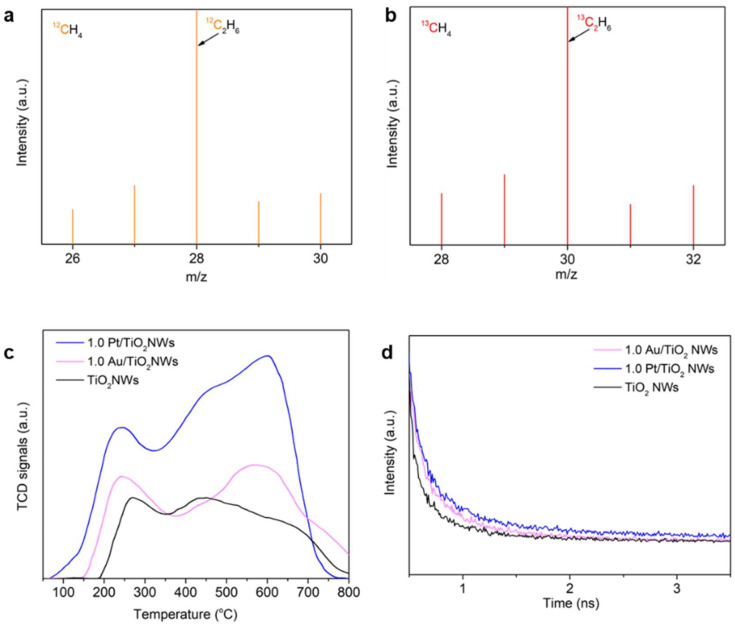
(**a**) Mass spectrum (*m*/*z* = 28–32) of gas products after 30 min light irradiation using 20 mg 1.0 Au/TiO_2_ NW catalyst and 92.56 KPa ^12^CH_4_. (**b**) Isotope-labelled mass spectrum (*m*/*z* = 28–32) of gas products under the same conditions using 92.56 KPa ^13^CH_4_ in photocatalytic oxidative coupling of methane reaction. (**c**) O_2_-TPD spectra of TiO_2_ NWs, 1.0 Au/TiO_2_ NWs, and 1.0 Pt/TiO_2_ NWs. (**d**) PL decay spectra of TiO_2_ NWs, 1.0 Au/TiO_2_ NWs, and 1.0 Pt/TiO_2_ NWs.

To further confirm that C_2_H_6_ was generated from CH₄, an isotope experiment was conducted using equal amounts of ^12^CH_4_ or ^13^CH_4_ as the reactant. The products were then analyzed using gas chromatography–mass spectrometry (GC-MS) (Figure 4a,b). As shown in Figure 4a, a peak at *m*/*z* = 28, attributed to the generation of ^12^C_2_H_6_, is clearly observed. When the reactant is replaced with ^13^CH_4_, the peak shifts from the lower mass–charge ratio (*m*/*z* = 28) to *m*/*z* = 30, corresponding to ^13^C_2_H_6_ (Figure 4b). This shift in the peak further confirms that C_2_H_6_ is indeed derived from the methane coupling reaction driven by photocatalysis.

According to previous work, the mild adsorption and activation of O_2_ play a decisive role in the selective oxidation of methane [29]. To investigate the adsorption and activation of O₂ on the photocatalysts in this work, temperature-programmed desorption (O₂-TPD) experiments were performed. This technique provides insights into the strength and nature of oxygen adsorption on the catalyst surface. There was only one broad O_2_ desorption peak presented on TiO_2_ NWs, 1.0 Au/TiO_2_ NWs, and 1.0 Pt/TiO_2_ NWs in the temperature range of 25 to 350 °C. The TiO_2_ NWs displayed a small amount of oxygen desorption, while 1.0 Au/TiO_2_ NWs and 1.0 Pt/TiO_2_ NWs exhibited substantial oxygen desorption, illustrating that Au and Pt improve the oxygen adsorption capacity of the catalyst (Figure 4c). For the TiO_2_ NW catalyst, the oxygen desorption occurred at 185 °C and the peak was at 268 °C. After Au loading, the oxygen desorption occurred at 142 °C and the peak was at 242 °C, shifted to the low-temperature range [30,31]. Unexpectedly, the Pt addition decreased the temperature of oxygen desorption further, starting at 64 °C and centered at 240 °C, suggesting that Pt could improve the mobility and activation of the O_2_ better than Au. This was easy to cause excessive activation of O_2_ on Pt/TiO_2_ NWs, which induced the deep oxidation of CH_4_. The mild activation of O_2_ by Au should contribute to desorption of reaction intermediates to promote the excellent selective oxidation of CH_4_ to C_2_H_6_.

In addition, oxygen molecules adsorbed on the surface of catalysts act as electron scavengers, capturing photogenerated electrons to form reactive oxygen species like superoxide anions (O_2_^−^). These superoxide anions drive the oxidation of methane, a key step in methane conversion. Compared to 1.0 Au/TiO_2_ NWs, the longer PL decay lifetime observed for 1.0 Pt/TiO_2_ NWs indicated that photoinduced electron–hole pairs were less likely to recombine in the Pt-loaded system. This suggested that more electrons were available for oxygen activation, enhancing the generation of reactive superoxide anions (Figure 4d). According to the Photoluminescence Spectroscopy data, the 1.0 Pt/TiO_2_ NWs also showed the highest separation efficiency of photogenerated carries (Figure S10). The excessive superoxide anions produced on the surface of 1.0 Pt/TiO_2_ NWs were highly reactive and induced the complete oxidation of methane to CO_2_, rather than the partial oxidation of CH_4_ to C_2_H_6_.

**Figure 5:**
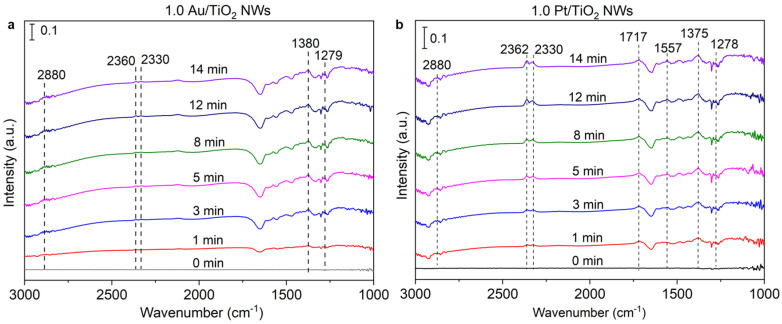
(**a**) In situ DRIFT spectroscopy characterization of 1.0 Au/TiO_2_ NWs. (**b**) In situ DRIFT spectroscopy characterization of 1.0 Pt/TiO_2_ NWs (in situ DRIFT spectra were test in reactant gas (CH_4_/air ratio, 69/1) under different light irradiation times).

In situ FTIR was employed to study the photocatalytic oxidization coupling of the methane reaction process on the Au/TiO_2_ NWs and Pt/TiO_2_ NWs. Methane was featured with typical IR vibration modes around 3015 cm^−1^ and 1305 cm^−1^ and multiple IR bands around 3015 cm^−1^ (Figure S11). The FTIR spectra of 1.0 Pt/TiO_2_ NWs (Figure 5b) showed that the band appeared at 2880 cm^−1^ attributed to the methoxy groups (CH_3_O_(a)_), and the intensity was much stronger than that of 1.0 Au/TiO_2_ NWs (Figure 5a). Additionally, lower-frequency bands in the range of 1717 cm^−1^ were associated with surface-bound formaldehyde species (CH_2_O_(a)_). The adsorbed formate (HCOO^−^) species were evidenced by the appearance of new bands around 1557 cm^−1^, attributed to the -COO- antisymmetric stretching mode, as well as a sharp increase in the band at approximately 1375 cm^−1^, which corresponds to the -COO- antisymmetric stretching mode [32,33]. These typical peaks further confirmed that HCOOH served as an intermediate in the methane conversion reaction over the 1.0 Pt/TiO_2_ NWs photocatalyst, and HCOOH was the important reaction intermediate for the deep oxidation of methane. The presence of carbon dioxide absorbed on the surface of 1.0 Pt/TiO_2_ NWs was verified by the rotational stretching bands observed in the range of 2362–2330 cm^−1^, and the intensity was more intensive than that of 1.0 Au/TiO_2_ NWs. These findings revealed that methane deep oxidation was the sole reaction occurring over the Pt/TiO_2_ NWs photocatalyst (•CH_3_ + O_2_^−^ → CH_2_O + OH^−^; CH_2_O + O_(s)_ → HCOOH_(a)_; HCOOH_(a)_ + h^+^ → CO_2_ + H^+^), while 1.0 Au/TiO_2_ NWs were more inclined to promote the OCM reaction (•CH_3_+ •CH_3_ → C_2_H_6_).

## 3. Materials and Methods

### 3.1. Material Synthesis

Preparation of anatase TiO_2_ nanowires: In a typical synthesis, 0.2 g of lithium acetate dihydrate (LiAc, FUJIFILM Wako Pure Chemical Corporation, Osaka, Japan) was dissolved in 10 mL of organic solvent containing N, N-dimethylformamide (DMF, FUJIFILM Wako Pure Chemical Corporation, Osaka, Japan, 99%), and acetic acid (HAc, FUJIFILM Wako Pure Chemical Corporation, Osaka, Japan, 100%). After the addition of 2 mL of titanium (IV) butoxide (TB, Sigma-Aldrich Japan K.K., Tokyo, Japan, ≥97%), the mixture solution was stirred for half an hour, then transferred into 50 mL Teflon-liner autoclave; after that, the autoclave was put into the oven for hydrothermal reaction at 200 °C for 20 h. Finally, the product was collected and washed thoroughly with ethanol several times, and then dried in a vacuum oven at 60 °C overnight. The mixed solvent with the ratio of DMF/HAc (*v*/*v*) that was used for the synthesis was 5/5 [22].

Preparation of noble metal loaded TiO_2_ nanowires: TiO_2_ nanowires (TiO_2_ NWs) produced by the above method were adopted for use. Synthesis of metal (Pt, Pt, Au, and Ag)/TiO_2_ photocatalysts: Cocatalysts (Pt, Pd, Au, and Ag) were loaded on the TiO_2_ NW photocatalysts via the NaBH_4_ reduction method. The precursors of cocatalysts were HAuCl_4_·4H_2_O (FUJIFILM Wako Pure Chemical Corporation, Osaka, Japan, 99.9%), AgNO_3_ (FUJIFILM Wako Pure Chemical Corporation, Osaka, Japan, 99.8%), Na_2_PdCl_4_ (Sigma-Aldrich Japan K.K., Tokyo, Japan, 99.99%), and H_2_PtCl_6_·6H_2_O (FUJIFILM Wako Pure Chemical Corporation, Osaka, Japan, 99.9%). Typically, as for the synthesis of 1 wt.% Au/TiO_2_ nanowire photocatalyst, 0.1 g of TiO_2_ NWs was dispersed into 100.0 mL deionized water by ultrasonication. Then, a certain amount of HAuCl_4_ (2.4 × 10^−2^ M) was added to the aqueous solution. After stirring for 30 min, a freshly prepared NaBH_4_ (FUJIFILM Wako Pure Chemical Corporation, Osaka, Japan, 99%) solution was dropwise added to the above mixed solution. After a further stirring for 30 min, the samples were collected by filtering, washed with deionized water several times, and dried for 12 h at 70 °C. The synthesis procedure of 0.1 wt.% Au/TiO_2_ nanowires, 0.5 wt.% Au/TiO_2_ nanowires, 2 wt.% Au/ TiO_2_ nanowires, 1 wt.% Ag/TiO_2_ nanowires, 1 wt.% Pd/TiO_2_ nanowires, and 1 wt.% Pt/TiO_2_ nanowires was similar to the above method, except for changing the corresponding noble metal source. In this work, these prepared photocatalysts of x wt.% M/TiO_2_ nanowires (x = 0.1, 0.5, 1.0, 2.0; M = Au, Ag, Pd, and Pt) were named as x M/TiO_2_ NWs.

### 3.2. Material Characterizations

The atomic structural information of the sample was determined by X-ray diffraction (XRD, PANalytical X’Pert PRO). High-resolution scanning TEM images were obtained using an FEI Talos F200s. The high-angle annular dark-field scanning transmission electron microscope (HAADF-STEM, Hitachi, Tokyo, Japan) and energy-dispersive X-ray spectroscopy (EDS) elemental mapping were recorded on an FEI Titan 80-300 microscope (Hitachi, Tokyo, Japan) equipped with a monochromator and a probe Cs corrector operated at 300 kV. Ultraviolet-visible spectroscopy (UV-vis, UV-2600 Shimadzu Corp., Kyoto, Japan) absorption spectra were recorded by means of the diffuse reflection mode using an Agilent Cary 60 (Agilent Technologies Inc., Santa Clara, CA, USA) UV-visible spectrometer. The surface chemical analysis was conducted on X-ray photoelectron spectroscopies (XPS, Escalab 250Xi, Thermal Fisher, Bedford, MA, USA). O_2_ temperature programmed desorption (O_2_-TPD) was conducted by AutoChem II 2920 (Micromeritics Instrument Corporation, Norcross, GA, USA). Additionally, 50 mg of catalysts was initially pretreated at 150 °C in Ar atmosphere for 1 h, followed by cooling down to room temperature. Then, the catalyst was exposed to a pure O_2_ environment for about 30 min in the system for adsorption. After that, the temperature was increased from 50 °C to 900 °C at a heating rate of 5 °C min^−1^ in helium (He) atmosphere.

### 3.3. Photocatalytic Oxidative Coupling of Methane (CH_4_)

Photocatalytic activities were evaluated by the production of ethane (C_2_H_6_) through the oxidative coupling of methane (OCM) under simulated solar irradiation without any sacrificial agent. Typically, 20 mg of catalyst was put on the surface of sample holder located in a quartz glass reaction cell. Before the irradiation, argon (Ar) gas was introduced in the reaction cell to remove air completely. Then, the system was fed with methane (purity, 99.9995%, Tokyo Gas Chemicals, Tokyo, Japan) and air (20 vol.% O_2_/N_2_, Tokyo Gas Chemicals, Tokyo, Japan) with a flowing rate at 69 mL min^−1^ and 1 mL min^−1^, respectively. A 300 W xenon arc lamp equipped with a colored glass filter (HA 30, HOYA, Tokyo, Japan) to remove all the infrared light was utilized as the light source with light intensity of 500 mW cm^−2^. A thermocouple was inserted into the reaction cell to directly detect the temperature around the photocatalyst. When the reaction was stable, the gas products were analyzed by gas chromatograph (GC-8A, Shimadzu Corp., Ar as carrier gas, TCD, Kyoto, Japan) equipped with a methanizer and flame ionization detector. C_2_H_6_ selectivity (%) = the mole of C_2_H_6_ × 100/the mole of (C_2_H_6_ + C_2_H_4_ + C_3_H_8_ + CO + CO_2_). C_2_–C_3_ selectivity (%) = the mole of (C_2_H_6_ + C_2_H_4_ + C_3_H_8_) × 100 / the mole of (C_2_H_6_ + C_2_H_4_ + C_3_H_8_ + CO + CO_2_).

The isotopic labeling test was conducted in a batch reactor: 200 mL ^13^C enriched CH_4_ (^13^C enrichment: >99 atom%, Tokyo Gas Chemicals, Tokyo, Japan) and 10 mL air (20 vol.% O_2_/N_2_, Tokyo Gas Chemicals, Tokyo, Japan) were introduced into the batch reactor after evacuation. The experiment was performed under light irradiation for 3 h. Then, the gas product was analyzed by chromatography–mass spectrometry (GC-MS).

## 4. Conclusions

In the photocatalytic OCM reaction, the 1.0 Au/TiO_2_ NWs demonstrated remarkable activity, particularly in terms of C_2_H_6_ production rate (4901 μmol g^−1^ h^−1^) and selectivity (70%), outperforming other noble-metal-loaded TiO_2_ NW catalysts. Compared to Pt/TiO_2_ NWs, Au/TiO_2_ NWs exhibited milder oxygen adsorption and activation properties, promoting selective methane coupling reactions, while the Pt seemed to be more active in oxidation catalysts fully oxidizing methane to CO_2_. Spectral and kinetic studies further revealed that Au enhanced the separation efficiency of photogenerated electrons and holes, and effectively promoted the appropriate amount of reactive oxygen species generation during the methane conversion process, avoiding excessive oxidation. Overall, the excellent performance of the Au/TiO_2_ NW catalysts in selective oxidation reactions demonstrates the crucial role of metal cocatalysts in optimizing photocatalytic performance, especially in controlling the capacity of oxygen adsorption and activation. This research not only offers a theoretical basis for developing efficient methane photocatalytic conversion catalysts, but also provides valuable insights for future photocatalyst design.

## Data Availability

Data are contained within the article and Supplementary Materials.

## Supplementary Materials

The following supporting information can be downloaded at: https://www.mdpi.com/article/10.3390/molecules30020206/s1, Figure S1: SEM images of TiO_2_ nanowires; Figure S2: STEM images of TiO_2_ nanowires; Figure S3: a, XRD patterns of TiO_2_ NWs loaded with Ag, Pd and Pt. b, XRD patterns of 0.1 Au/ TiO_2_ NWs, 0.5 Au/ TiO_2_ NWs, and 2.0 Au/TiO_2_ NWs; Figure S4: XPS patterns of 1.0 Au/TiO_2_ NWs; Figure S5: a, XPS spectra of Ti 2p for TiO_2_ NWs. b, XPS spectra of Ti 2p for 1.0 Au/TiO_2_ NWs; Figure S6: a–e, Adsorption-desorption for TiO_2_ NWs loaded with different amount of Au. f, Adsorption-desorption for TiO_2_ NWs loaded with Pt. The insert of a, b, c, d and e are pore size distribution for TiO_2_ NWs loaded with different amount of Au. The insert of the f is pore size distribution for TiO_2_ NWs loaded with Pt; Figure S7: XRD patterns of 1.0 Au/TiO_2_ NWs, 1.0 Au/TiO_2_ anatase (nanoparticle),1.0 Au/TiO_2_ Rutile (nanoparticle), 1.0 Au/P25; Figure S8: Photocatalytic OCM performance over 1.0 Au/TiO_2_ NWs, 1.0 Au/TiO_2_ (Anatase, nanoparticle),1.0 Au/P25 and 1.0 Au/ TiO_2_ (Rutile); Figure S9: UV–vis diffuse absorption spectrum of 1.0 Au/TiO_2_ NWs and 2.0 Au/TiO_2_ NWs; Figure S10: PL spectra of TiO_2_ NWs, 1.0 Au/TiO_2_ NWs and 1.0 Pt/ TiO_2_ NWs; Figure S11: FTIR spectra of CH_4_; Table S1: The products and selectivity of TiO_2_ NWs with different amounts of Au in OCM reaction.

## References

[1] Olivos-Suarez A.I., Szecsenyi A., Hensen E.J.M., Ruiz-Martinez J., Pidko E.A., Gascon J. (2016). Strategies for the Direct Catalytic Valorization of Methane Using Heterogeneous Catalysis: Challenges and Opportunities. ACS Catal..

[2] Li Q., Ouyang Y., Li H., Wang L., Zeng J. (2022). Photocatalytic Conversion of Methane: Recent Advancements and Prospects. Angew. Chem. Int. Edit..

[3] Li X., Wang C., Tang J. (2022). Methane transformation by photocatalysis. Nat. Rev. Mater..

[4] Schwach P., Pan X., Bao X. (2017). Direct Conversion of Methane to Value-Added Chemicals over Heterogeneous Catalysts: Challenges and Prospects. Chem. Rev..

[5] Yuliati L., Yoshida H. (2008). Photocatalytic conversion of methane. Chem. Soc. Rev..

[6] Tang Y., Li Y., Tao F. (2022). Activation and catalytic transformation of methane under mild conditions. Chem. Soc. Rev..

[7] Song H., Meng X.G., Wang Z.J., Liu H.M., Ye J.H. (2019). Solar-Energy-Mediated Methane Conversion. Joule.

[8] Lunsford J.H. (1995). The Catalytic Oxidative Coupling of Methane. Angew. Chem. Int. Edit..

[9] Wang P., Zhao G., Wang Y., Lu Y. (2017). MnTiO_3_-driven low-temperature oxidative coupling of methane over TiO_2_-doped Mn_2_O_3_-Na_2_WO_4_/SiO_2_ catalyst. Sci. Adv..

[10] Li L., Li G.-D., Yan C., Mu X.-Y., Pan X.-L., Zou X.-X., Wang K.-X., Chen J.-S. (2011). Efficient Sunlight-Driven Dehydrogenative Coupling of Methane to Ethane over a Zn^+^-Modified Zeolite. Angew. Chem. Int. Edit..

[11] Chen X., Li Y., Pan X., Cortie D., Huang X., Yi Z. (2016). Photocatalytic oxidation of methane over silver decorated zinc oxide nanocatalysts. Nat. Commun..

[12] Xie J.J., Jin R.X., Li A., Bi Y.P., Ruan Q.S., Deng Y.C., Zhang Y.J., Yao S.Y., Sankar G., Ma D. (2018). Highly selective oxidation of methane to methanol at ambient conditions by titanium dioxide-supported iron species. Nat. Catal..

[13] Wang Y., Zhang Y., Liu Y., Wu Z. (2023). Photocatalytic Oxidative Coupling of Methane to Ethane Using Water and Oxygen on Ag_3_PO_4_-ZnO. Environ. Sci. Technol..

[14] Zhang W., Fu C., Low J., Duan D., Ma J., Jiang W., Chen Y., Liu H., Qi Z., Long R. (2022). High-performance photocatalytic nonoxidative conversion of methane to ethane and hydrogen by heteroatoms-engineered TiO_2_. Nat. Commun..

[15] Meng L., Chen Z., Ma Z., He S., Hou Y., Li H.-H., Yuan R., Huang X.-H., Wang X., Wang X. (2018). Gold plasmon-induced photocatalytic dehydrogenative coupling of methane to ethane on polar oxide surfaces. Energy Environ. Sci..

[16] Hu D., Dong C., Belhout S., Shetty S., Ng H., Brasseur P., Bezerra L.S., Tayeb K.B., Simon P., Addad A. (2023). Roles of titania and plasmonic gold nanoparticles of different sizes in photocatalytic methane coupling at room temperature. Mater. Today Energy.

[17] Wang C., Li X., Ren Y., Jiao H., Wang F.R., Tang J. (2023). Synergy of Ag and AgBr in a pressurized flow reactor for selective photocatalytic oxidative coupling of methane. ACS Catal..

[18] Yu X., Zholobenko V.L., Moldovan S., Hu D., Wu D., Ordomsky V.V., Khodakov A.Y. (2020). Stoichiometric methane conversion to ethane using photochemical looping at ambient temperature. Nat. Energy.

[19] Cao H., Liu F., Tai Y., Wang W., Li X., Li P., Zhao H., Xia Y., Wang S. (2023). Promoting photocatalytic performance of TiO_2_ nanomaterials by structural and electronic modulation. Chem. Eng. J..

[20] Wang X., Li Z., Shi J., Yu Y. (2014). One-dimensional titanium dioxide nanomaterials: Nanowires, nanorods, and nanobelts. Chem. Rev..

[21] Nakata K., Fujishima A. (2012). TiO_2_ photocatalysis: Design and applications. J. Photochem. Photobiol. C.

[22] Wu H.B., Hng H.H., Lou X.W. (2012). Direct Synthesis of Anatase TiO_2_ Nanowires with Enhanced Photocatalytic Activity. Adv. Mater..

[23] Gong M.C., Zhou J.L., Xu Z.H., Chen Y.Q., Chen Y. (1995). Study on the oxidative coupling of methane: XRD and XPS study of TiO_2_-based catalysts promoted by different additives. Catal. Today.

[24] Huo S., Wu Y., Zhao C., Yu F., Fang J., Yang Y. (2020). Core–Shell TiO_2_@Au_25_/TiO_2_ Nanowire Arrays Photoanode for Efficient Photoelectrochemical Full Water Splitting. Ind. Eng. Chem. Res..

[25] Wu N., Wang J., Tafen D.N., Wang H., Zheng J.-G., Lewis J.P., Liu X., Leonard S.S., Manivannan A. (2010). Shape-Enhanced Photocatalytic Activity of Single-Crystalline Anatase TiO_2_ (101) Nanobelts. J. Am. Chem. Soc..

[26] Li X.Y., Xie J.J., Rao H., Wang C., Tang J.W. (2020). Platinum- and CuOx-Decorated TiO_2_ Photocatalyst for Oxidative Coupling of Methane to C_2_ Hydrocarbons in a Flow Reactor. Angew. Chem. Int. Edit..

[27] Saito H., Sato H., Higashi T., Sugimoto T. (2023). Beyond reduction cocatalysts: Critical role of metal cocatalysts in photocatalytic oxidation of methane with water. Angew. Chem. Int. Edit..

[28] Jiang W.B., Low J.X., Mao K.K., Duan D.L., Chen S.M., Liu W., Pao C.W., Ma J., Sang S.K., Shu C. (2021). Pd-Modified ZnO–Au Enabling Alkoxy Intermediates Formation and Dehydrogenation for Photocatalytic Conversion of Methane to Ethylene. J. Am. Chem. Soc..

[29] Song S., Song H., Li L., Wang S., Chu W., Peng K., Meng X., Wang Q., Deng B., Liu Q. (2021). A selective Au-ZnO/TiO_2_ hybrid photocatalyst for oxidative coupling of methane to ethane with dioxygen. Nat. Catal..

[30] Zhang C., Li Y., Wang Y., He H. (2014). Sodium-promoted Pd/TiO_2_ for catalytic oxidation of formaldehyde at ambient temperature. Environ. Sci. Technol..

[31] Iwamoto M., Yoda Y., Yamazoe N., Seiyama T. (1978). Study of metal oxide catalysts by temperature programmed desorption. 4. Oxygen adsorption on various metal oxides. J. Phys. Chem. C.

[32] Kang H., Zhu L., Li S., Yu S., Niu Y., Zhang B., Chu W., Liu X., Perathoner S., Centi G. (2023). Generation of oxide surface patches promoting H-spillover in Ru/(TiOx)MnO catalysts enables CO_2_ reduction to CO. Nat. Catal..

[33] Kang H., Ma L., Li S., Chen X., Chu W., Zhang R., Perathoner S., Centi G., Liu Y. (2024). Oxygen vacancy-dependent chemical intermediates on Ru/MnO catalysts dictate the selectivity of CO_2_ reduction. Appl. Catal. B-Environ Energy.

