# Supplementary material for: Enhanced Photocatalytic Oxidative Coupling of Methane over Metal-Loaded TiO2 Nanowires
Source: Molecules. 2025 Jan 7;30(2):206. doi: 10.3390/molecules30020206 (PMC11767848; doi:10.3390/molecules30020206)
Supplement: Supplementary file 1 [file molecules-30-00206-s001.zip › molecules-3389335-supplementary.pdf]

Supporting Information

Enhanced Photocatalytic Oxidative Coupling of Methane Over Metal-Loaded TiO_2 Nanowires

Shuang Song [1], Jiongcan Xiang [1], Hui Kang [2,*] and Fengming Yang [3,*]

1. Institute of Fundamental and Frontier Sciences, University of Electronic Science and Technology of China, Sichuan, Chengdu 610054, China.
2. Institute of Advanced Study, Chengdu University, Chengdu 610106, China
3. College of Computer Science and Cyber Security (Pilot Software College), Chengdu University of Technology, Chengdu 610059, China.

* Correspondence: kanghui@cdu.edu.cn (H.K.); fmyang2020@163.com (F.Y.)

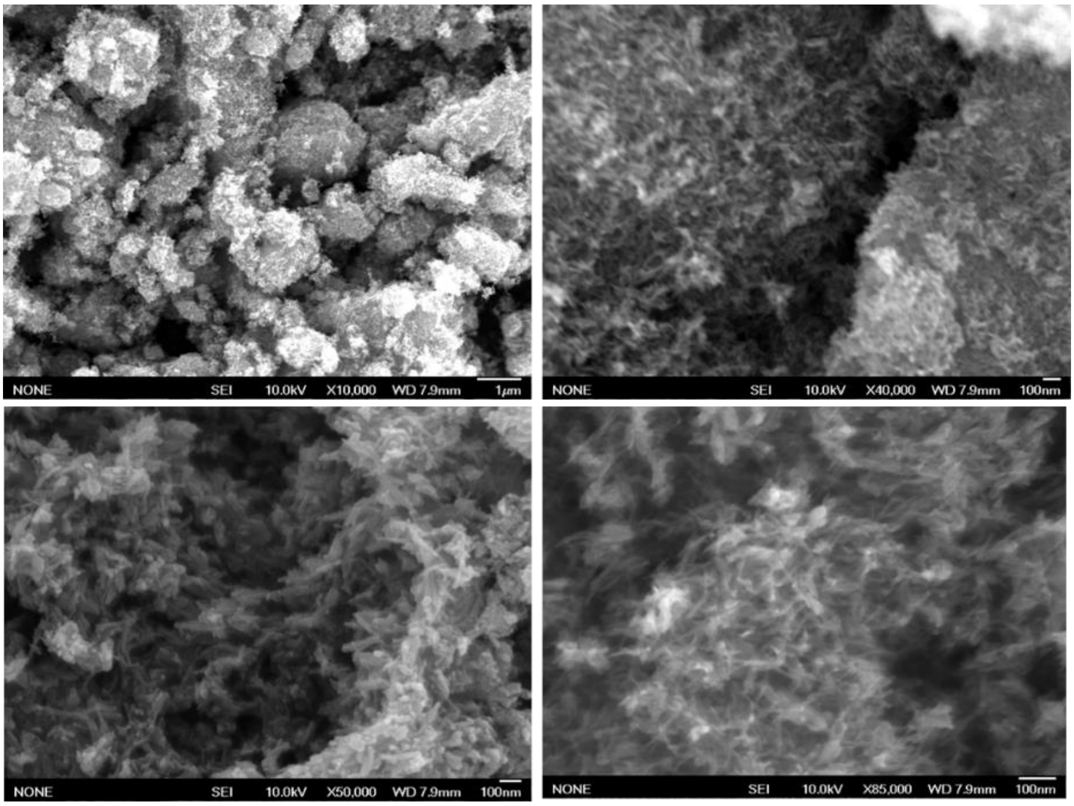

Figure S1. SEM images of TiO_2 nanowires.

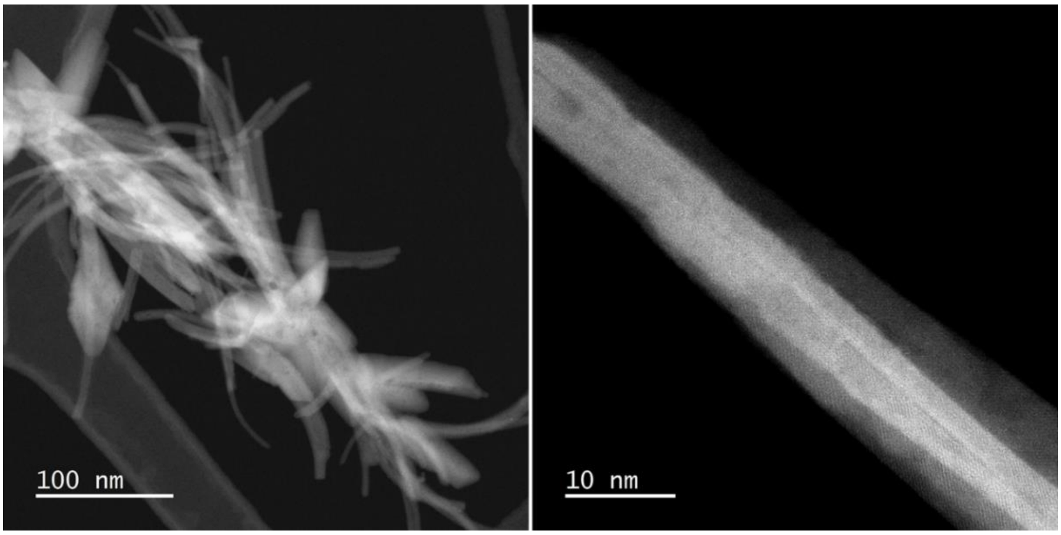

Figure S2. STEM images of TiO_2 nanowires.

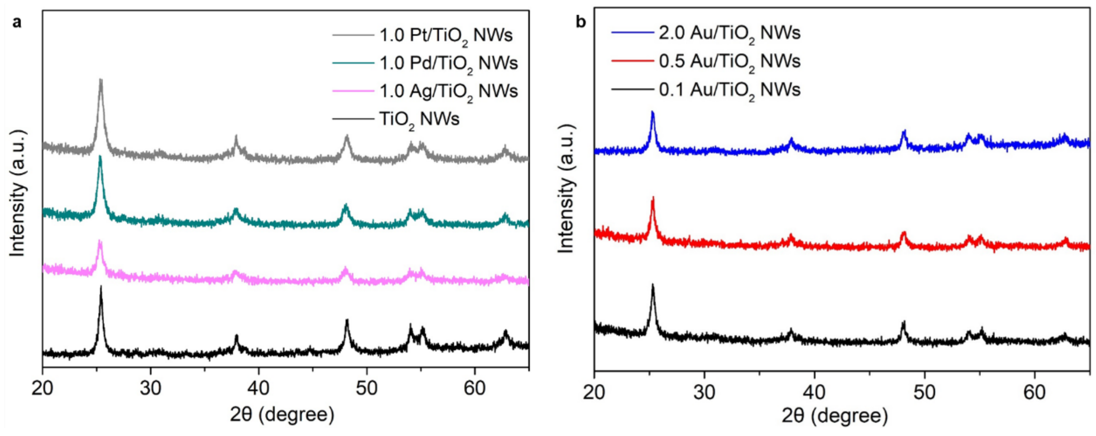

Figure S3. a, XRD patterns of TiO_2 NWs loaded with Ag, Pd and Pt. b, XRD patterns of 0.1 Au/ TiO_2 NWs, 0.5 Au/ TiO_2 NWs, and 2.0 Au/TiO_2 NWs.

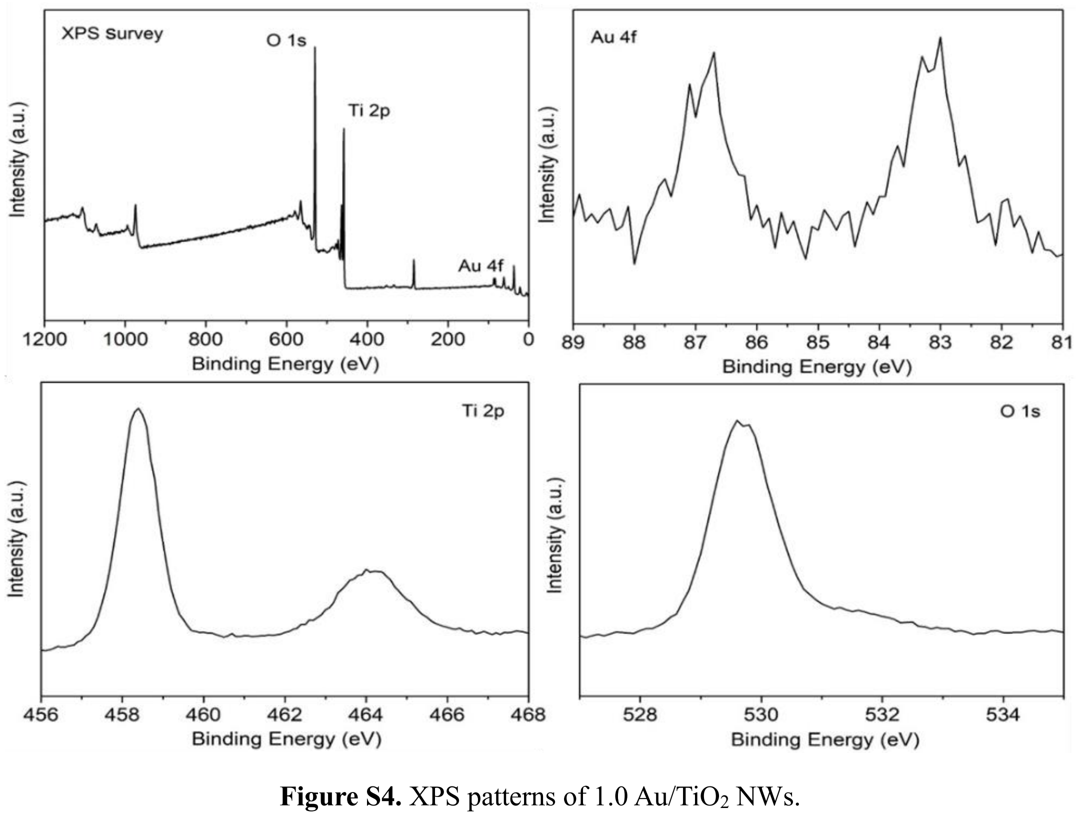

Figure S4. XPS patterns of 1.0 Au/TiO_2 NWs.

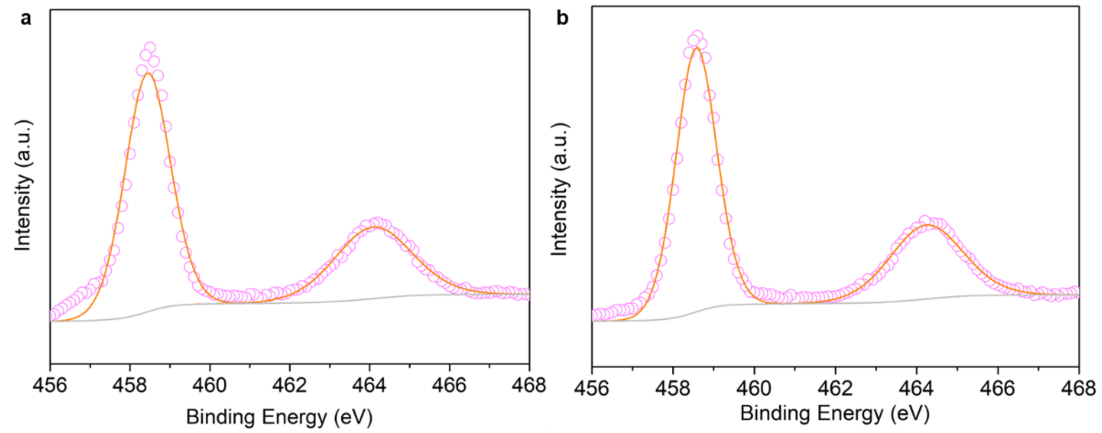

Figure S5. a**,** XPS spectra of Ti 2p for TiO_2 NWs. b**,** XPS spectra of Ti 2p for 1.0 Au/TiO_2 NWs.

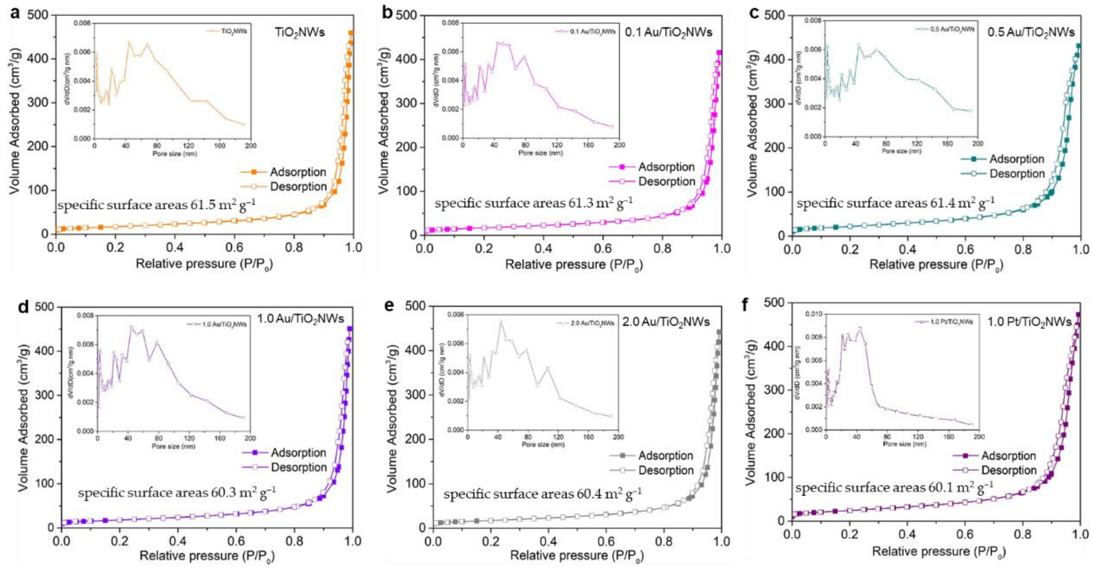

Figure S6. a-e**,** Adsorption-desorption for TiO_2 NWs loaded with different amount of Au. f, Adsorption-desorption for TiO_2 NWs loaded with Pt. The insert of a, b, c, d and e are pore size distribution for TiO_2 NWs loaded with different amount of Au. The insert of the f is pore size distribution for TiO_2 NWs loaded with Pt.

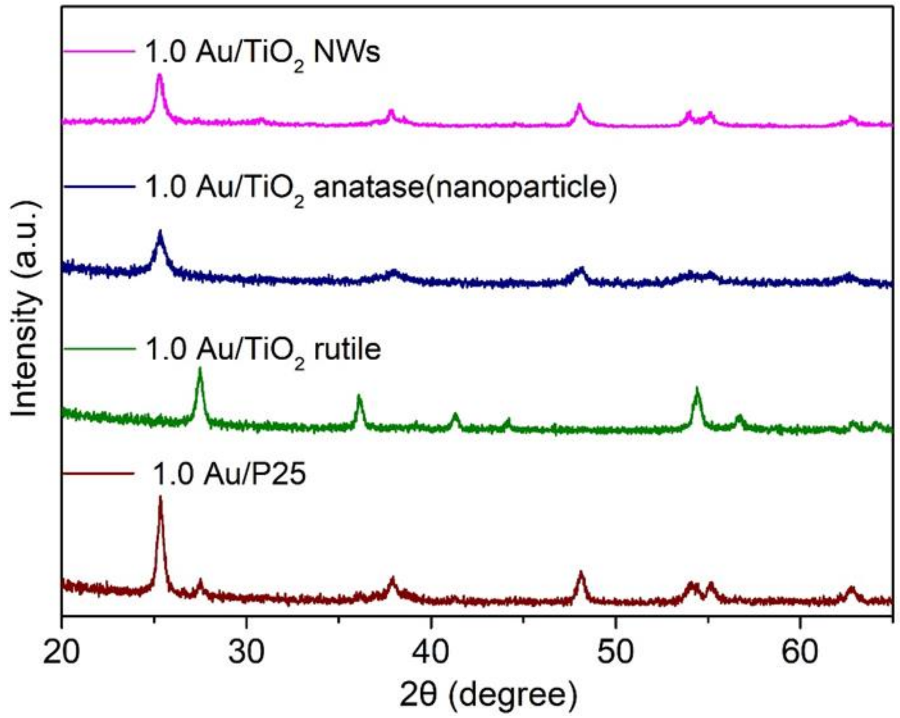

Figure S7. XRD patterns of 1.0 Au/TiO_2 NWs, 1.0 Au/TiO_2 anatase (nanoparticle),1.0 Au/TiO_2 Rutile (nanoparticle), 1.0 Au/P25.

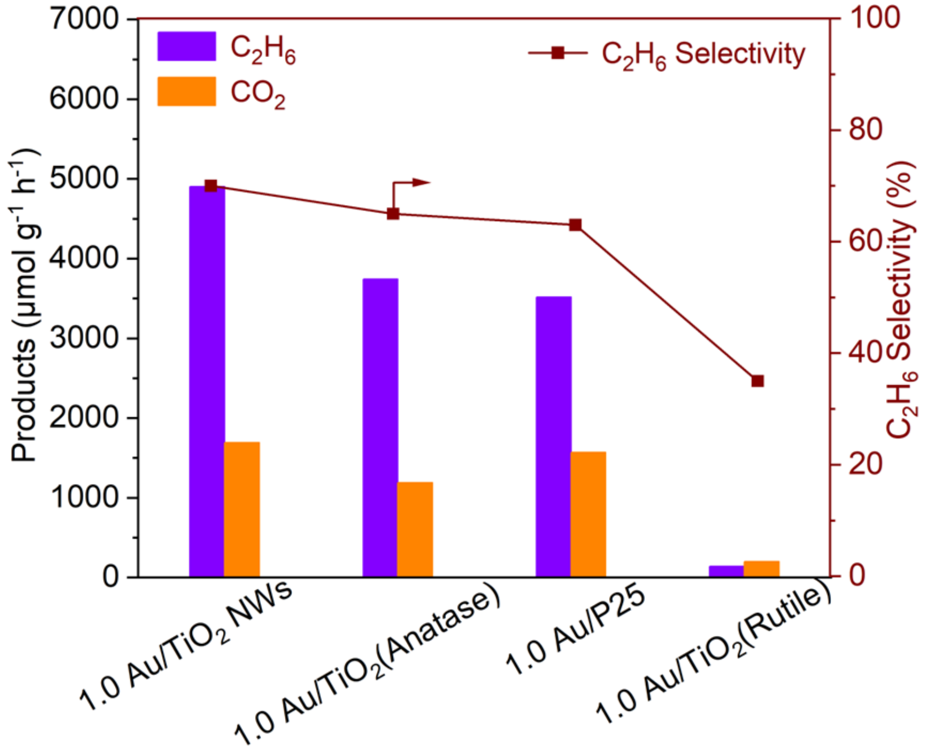

Figure S8. Photocatalytic OCM performance over 1.0 Au/TiO_2 NWs, 1.0 Au/TiO_2 (Anatase, nanoparticle),1.0 Au/P25 and 1.0 Au/ TiO_2 (Rutile).

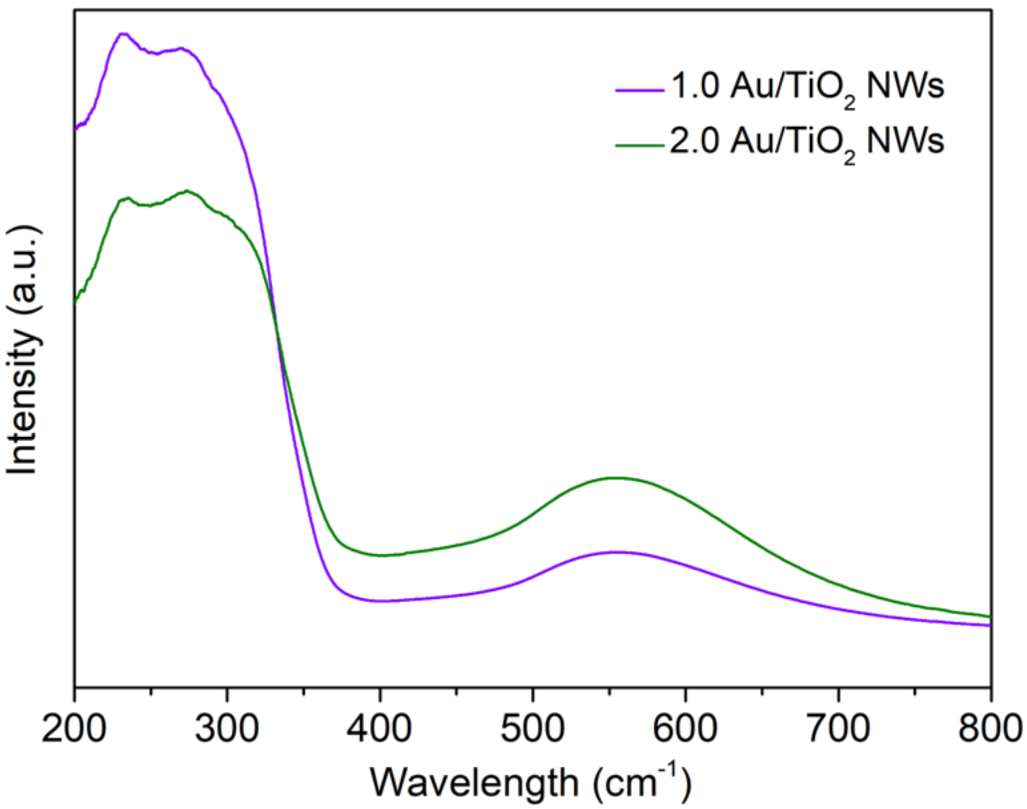

Figure S9. UV–vis diffuse absorption spectrum of 1.0 Au/TiO_2 NWs and 2.0 Au/TiO_2 NWs.

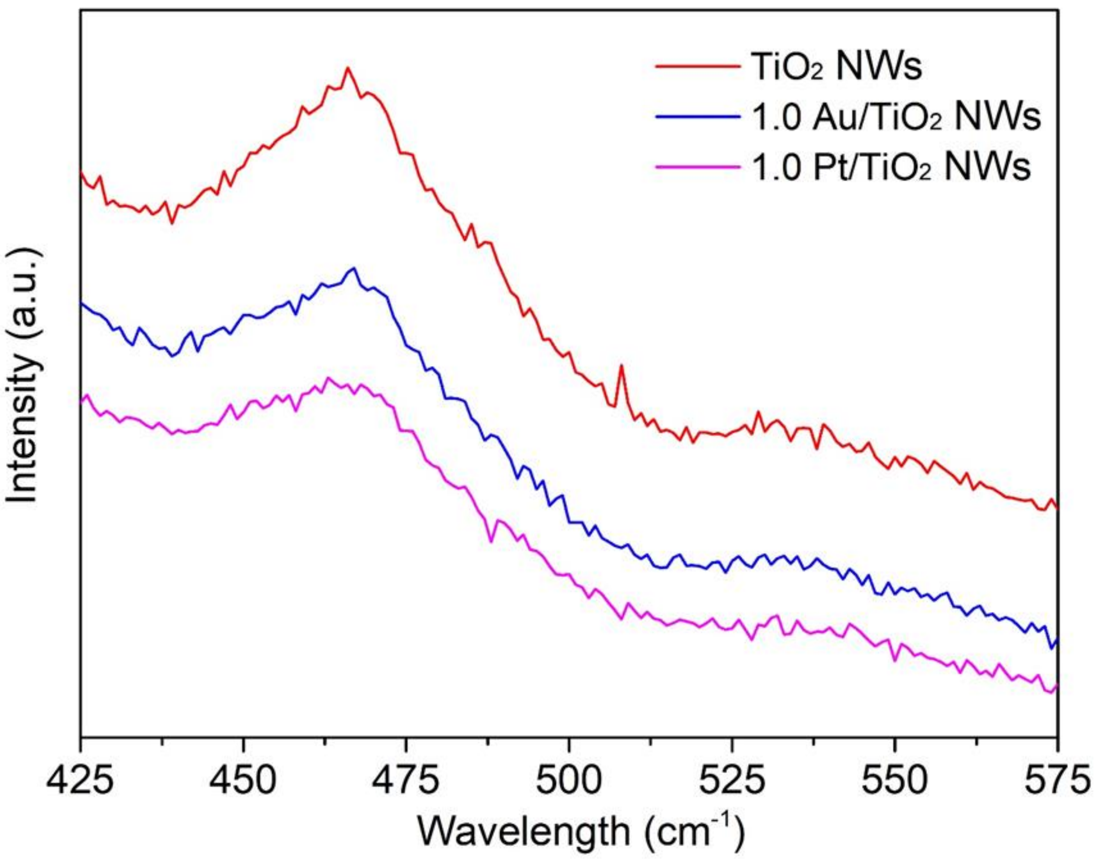

Figure S10. PL spectra of TiO_2 NWs, 1.0 Au/TiO_2 NWs and 1.0 Pt/ TiO_2 NWs.

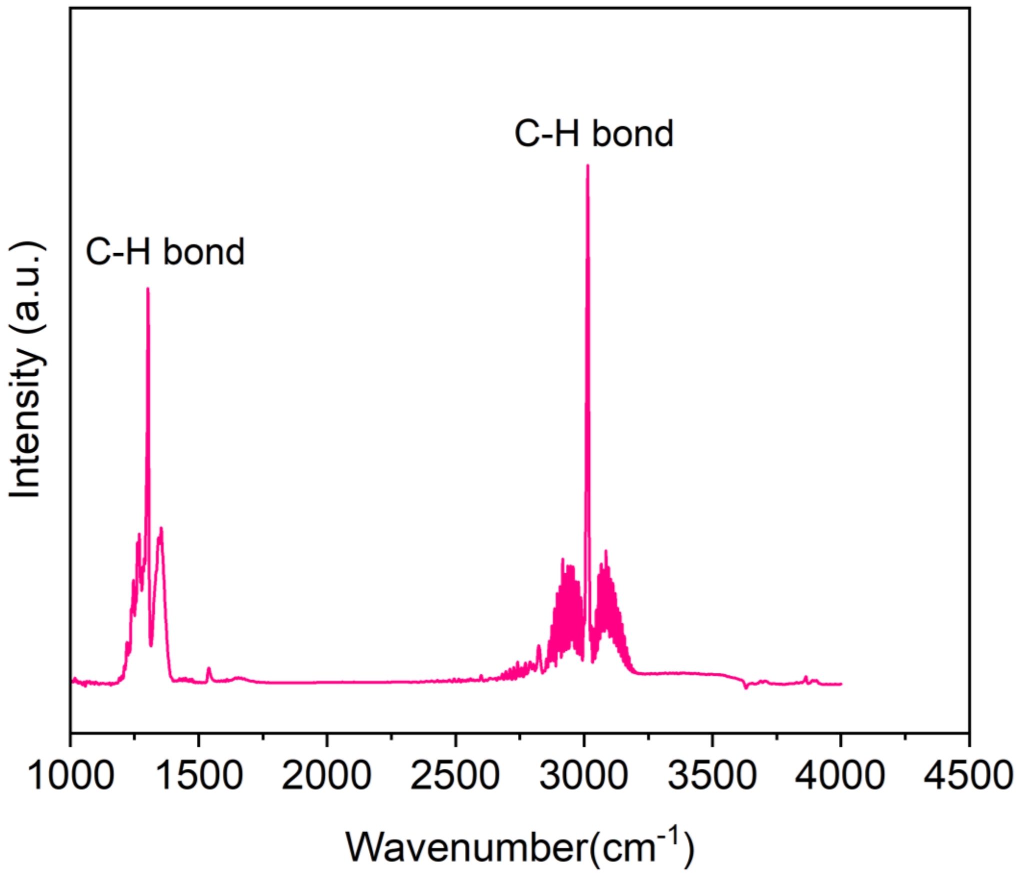

Figure S11. FTIR spectra of CH_4.

Table S1. The products and selectivity of TiO_2 NWs with different amounts of Au in OCM reaction.

Catalyst	Amount of product (μmol $g^{-1}h^{-1}$)					C_2-C_3 product (μmol $g^{-1}h^{-1}$)	All products (μmol $g^{-1}h^{-1}$)	C_2H_6 Selectivity (%)	C_2-C_3 selectivity (%)
	C_2H_6	C_2H_4	C_3H_8	CO	CO_2				
TiO_2 NWs	0	0	0	0	500	0	500	0	0
0.1 Au/TiO_2 NWs	2073	0	93	50	1144	2166	3360	61	64
0.5 Au/TiO_2 NWs	3246	0	287	28	1266	3533	4827	67	73
1.0 Au/TiO_2 NWs	4901	15	351	15	1692	5267	6988	70	75
2.0 Au/TiO_2 NWs	5444	26	378	0	1840	5848	7658	71	76
